# Supplementary material for: The Alteration of Circulating Invariant Natural Killer T, γδT, and Natural Killer Cells after Ischemic Stroke in Relation to Clinical Outcomes: A Prospective Case–Control Study
Source: Cells. 2024 Aug 22;13(16):1401. doi: 10.3390/cells13161401 (PMC11352391; doi:10.3390/cells13161401)
Supplement: Supplementary file 1 [file cells-13-01401-s001.zip › Table S1.pdf]

Table S1. Antibodies used in the study for cell immunophenotyping

Antibody	Fluorochrome	Clone	Source
CD3	APC-Cy7	UCH-1	BioLegend®, USA
CD4	PeCy7	SK3	BioLegend®, USA
CD8	PerCp	SK1	BioLegend®, USA
CD56	PerCP	A019D5	BioLegend®, USA
Vα24-Jα18 (iNKT)	APC	6B11	BioLegend®, USA
TCRγδ	APC	B1	BD Biosciences®, USA
TCR Vδ1	FITC	TS8.2	ThermoFisher Scientific®, USA
TCR Vδ2	Pe	B6	BioLegend®, USA
