# Supplementary material for: The Alteration of Circulating Invariant Natural Killer T, γδT, and Natural Killer Cells after Ischemic Stroke in Relation to Clinical Outcomes: A Prospective Case–Control Study
Source: Cells. 2024 Aug 22;13(16):1401. doi: 10.3390/cells13161401 (PMC11352391; doi:10.3390/cells13161401)
Supplement: Supplementary file 1 [file cells-13-01401-s001.zip › Table S2.pdf]

Table S2. Neurological Assessment Scale National Institute of Health Stroke Scale (NIHSS).

**1a**	**Level of Consciousness**	0 Alert<br>1 Not alert, but arousable with minimal stimulation<br>2 Not alert, requires repeated stimulation to attend<br>3 Coma
**1b**	**LOC Questions**	0 Answers both correctly<br>1 Answers one correctly<br>2 Answers neither correctly
**1c**	**LOC Commands**	0 Obeys both correctly<br>1 Obeys one correctly<br>2 Performs neither correctly
**2**	**Extraocular Movements**	0 Normal<br>1 Partial gaze palsy<br>2 Forced deviation or total gaze paresis not overcome by cephalic maneuver
**3**	**Visual Fields**	0 No visual loss<br>1 Partial hemianopsia<br>2 Complete hemianopsia<br>3 Bilateral hemianopsia (blind including cortical blindness)
**4**	**Facial Paresis**	0 Normal symmetrical movement<br>1 Minor paralysis (asymmetry on smiling, flattened nasolabial fold)<br>2 Partial paralysis (total or near total lower face paralysis)<br>3 Complete paralysis of one or both sides (upper and lower)
**5**	**Motor Arm**<br>**5A Right Arm**<br>**5B Left Arm**	0 Normal (extends arm 90° [or 45°] for 10 seconds without drift)<br>1 Drift<br>2 Some effort against gravity<br>3 No effort against gravity<br>4 No movement<br>9 Unstable (Joint fused or limb amputated)
**6**	**Motor Leg**<br>**6A Right Leg**<br>**6B Left Leg**	0 Normal<br>1 Drift<br>2 Some effort against gravity<br>3 No effort against gravity<br>4 No movement<br>9 Unstable (Joint fused<br>or limb amputated)
**7**	**Limb Ataxia**	0 No ataxia<br>1 Present in one limb<br>2 Present in two limbs
**8**	**Sensory** (Use pin prick to test<br>arms, legs, trunk and face – compare sides)	0 Normal<br>1 Mild to moderate decrease in sensation<br>2 Severe to total sensory loss
**9**	**Language** (describe picture, name items, read sentences)	0 No aphasia<br>1 Mild to moderate aphasia<br>2 Severe aphasia<br>3 Mute
**10**	**Dysarthria** (read several words)	0 Normal articulation<br>1 Mild to moderate slurring of words<br>2 Near unintelligible or unable to speak

		9 Intubated or other physical barrier
**11**	**Neglect**	0 No neglect<br>1 Inattention or extinction to bilateral simultaneous stimulation in one of the sensory modalities<br>2 Severe hemi−inattention or hemi−inattention to more than one modality
