# Supplementary figures and images for: The Alteration of Circulating Invariant Natural Killer T, γδT, and Natural Killer Cells after Ischemic Stroke in Relation to Clinical Outcomes: A Prospective Case–Control Study
Source: Cells. 2024 Aug 22;13(16):1401. doi: 10.3390/cells13161401 (PMC11352391; doi:10.3390/cells13161401)

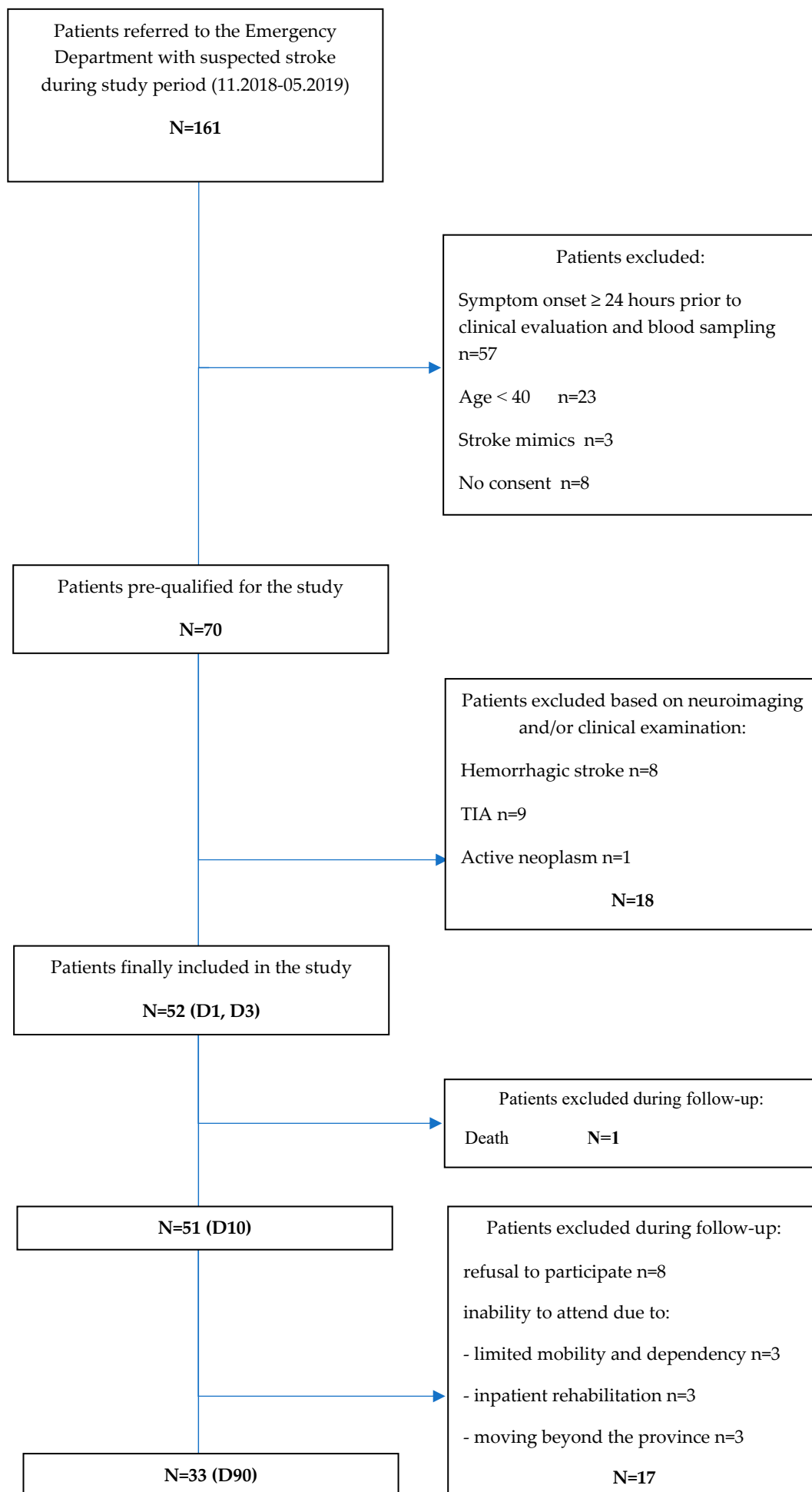

**Figure S1.** Flow chart of participant recruitment.

Supplement: Supplementary file 1 [file cells-13-01401-s001.zip › Figure S1.pdf]
